# Perfluorooctanesulfonic Acid Alters Pro-Cancer Phenotypes and Metabolic and Transcriptional Signatures in Testicular Germ Cell Tumors

**DOI:** 10.3390/toxics12040232

**Published:** 2024-03-22

**Authors:** Raya I. Boyd, Doha Shokry, Zeeshan Fazal, Brayden C. Rennels, Sarah J. Freemantle, Michael R. La Frano, Gail S. Prins, Zeynep Madak Erdogan, Joseph Irudayaraj, Ratnakar Singh, Michael J. Spinella

**Affiliations:** 1 Department of Comparative Biosciences, University of Illinois Urbana-Champaign, 2001 South Lincoln Avenue, Urbana, IL 61801, USA; rayaib2@illinois.edu (R.I.B.); dshokry@illinois.edu (D.S.); zeeshan.fazal1986@gmail.com (Z.F.); rennels3@illinois.edu (B.C.R.); sarahf@illinois.edu (S.J.F.); 2 Roy J. Carver Biotechnology Center, University of Illinois Urbana-Champaign, Urbana, IL 61801, USA; mlafrano@illinois.edu; 3 Departments of Urology, Pathology and Physiology, College of Medicine and Chicago Center for Health and Environment, University of Illinois Chicago, Chicago, IL 60612, USA; gprins@uic.edu; 4 Department of Food Science and Human Nutrition, Division of Nutritional Sciences, University of Illinois Urbana-Champaign, Urbana, IL 61801, USA; zmadake2@illinois.edu; 5 Carl R. Woese Institute for Genomic Biology, University of Illinois Urbana-Champaign, Urbana, IL 61801, USA; jirudaya@illinois.edu; 6 Cancer Center of Illinois, University of Illinois Urbana-Champaign, Urbana, IL 61801, USA; 7 Department of Bioengineering, University of Illinois, Urbana-Champaign, Urbana, IL 61801, USA

**Keywords:** PFOS, testicular cancer, metabolomics, transcriptomics, H3K27me3, fatty acid metabolism, steroid synthesis, HQ-115, LiTFSI, GenX

## Abstract

The potential effects of poly- and perfluoroalkyl substances (PFAS) are a recently emergent human and environmental health concern. There is a consistent link between PFAS exposure and cancer, but the mechanisms are poorly understood. Although epidemiological evidence supporting PFAS exposure and cancer in general is conflicting, there is relatively strong evidence linking PFAS and testicular germ cell tumors (TGCTs). However, no mechanistic studies have been performed to date concerning PFAS and TGCTs. In this report, the effects of the legacy PFAS perfluorooctanesulfonic acid (PFOS) and the newer “clean energy” PFAS lithium bis(trifluoromethylsulfonyl)imide (LiTFSi, called HQ-115), on the tumorigenicity of TGCTs in mice, TGCT cell survival, and metabolite production, as well as gene regulation were investigated. In vitro, the proliferation and survival of both chemo-sensitive and -resistant TGCT cells were minimally affected by a wide range of PFOS and HQ-115 concentrations. However, both chemicals promoted the growth of TGCT cells in mouse xenografts at doses consistent with human exposure but induced minimal acute toxicity, as assessed by total body, kidney, and testis weight. PFOS, but not HQ-115, increased liver weight. Transcriptomic alterations of PFOS-exposed normal mouse testes were dominated by cancer-related pathways and gene expression alterations associated with the H3K27me3 polycomb pathway and DNA methylation, epigenetic pathways that were previously showed to be critical for the survival of TGCT cells after cisplatin-based chemotherapy. Similar patterns of PFOS-mediated gene expression occurred in PFOS-exposed cells in vitro. Metabolomic studies revealed that PFOS also altered metabolites associated with steroid biosynthesis and fatty acid metabolism in TGCT cells, consistent with the proposed ability of PFAS to mimic fatty acid-based ligands controlling lipid metabolism and the proposed role of PFAS as endocrine disrupters. Our data, is the first cell and animal based study on PFAS in TGCTs, support a pro-tumorigenic effect of PFAS on TGCT biology and suggests epigenetic, metabolic, and endocrine disruption as potential mechanisms of action that are consistent with the non-mutagenic nature of the PFAS class.

## 1. Introduction

Poly- and perfluoroalkyl substances (PFAS) are industrial chemicals that contain at least one fluorocarbon group and are used in various applications, including water repellants, fire-fighting foams, and food packaging [1]. The production of PFAS chemicals started in the mid-1900s and has been in continual use [1,2]. PFAS are widely present and persist in the environment, bioaccumulate in organisms, and are associated with several negative health outcomes, including increased serum cholesterol, adverse birth outcomes, and an increased risk of cancer [3,4,5,6,7,8,9]. Legacy PFAS perfluorooctanoic acid (PFOA) and perfluorooctanesulfonic acid (PFOS) are suspected carcinogens [7,8]. PFOA is a group 2B carcinogen, while PFOS and the newer, shorter chain PFAS hexafluoropropylene oxide (GenX) are EPA-classified as potentially carcinogenic [10,11,12]. However, there is no evidence that PFAS are mutagenic or cause direct DNA damage, suggesting that PFAS carcinogenic effects are likely due to cell signaling or epigenetic mechanisms [13]. PFAS are also produced and environmentally present as bioproducts of the clean energy sector and include chemicals associated with windmill turbines, solar cell components, semi-conductors, and lithium-ion batteries [14]. The health effects of “clean energy” PFAS, including lithium bis(trifluoromethylsulfonyl)imide (LiTFSI, commonly referred to as HQ-115), are largely unknown.

Testicular germ cell tumors (TGCTs) are the most common cancers among males aged 15 to 45, and are a disease of developmental origin arising from aberrant primordial germ cells/gonocytes in utero [15]. TGCTs have very few mutations [16]. This suggests that epigenetic mechanisms play an important role in the etiology and biology of TGCTs [15,17]. Epidemiologic studies have indicated that the fetal gonads may be especially sensitive to pro-estrogenic and anti-androgenic insults [18,19,20,21]. Hence, TGCT etiology matches well with some of the most studied mechanisms of action of PFAS, namely, epigenetics and endocrine disruption (reviewed in [7,22]). Further, testicular cancer incidence rates have substantially increased since the introduction of industrial chemicals, including PFAS [23].

There is epidemiologic evidence linking TGCTs to PFAS exposure. However, there is no research on the mechanisms by which PFAS may trigger TGCTs [24,25,26,27,28,29,30]. In this report, we investigated the effects of PFOS and HQ-115 on the tumorigenicity of TGCTs in mice, TGCT cell survival and metabolite production, as well as gene regulation. Both PFOS and HQ-115 promoted the in vivo growth of TGCT cells that was associated with transcriptomic alterations in several cancer-related pathways, including the H3K27me3 polycomb pathway and DNA methylation. Metabolomic studies revealed that PFOS also altered metabolites associated with steroid biosynthesis and fatty acid metabolism in TGCT cells, consistent with the proposed ability of PFAS to mimic fatty acid-based ligands controlling lipid metabolism and the proposed role of PFAS as endocrine disrupters. Our data support a pro-tumorigenic effect of PFAS on TGCT biology and suggest epigenetic, metabolic, and endocrine disruption as potential mechanisms of action that are consistent with the non-mutagenic nature of the PFAS class.

## 2. Materials and Methods

### 2.1. Cell Proliferation/Survival Assays

**Figure 1:**
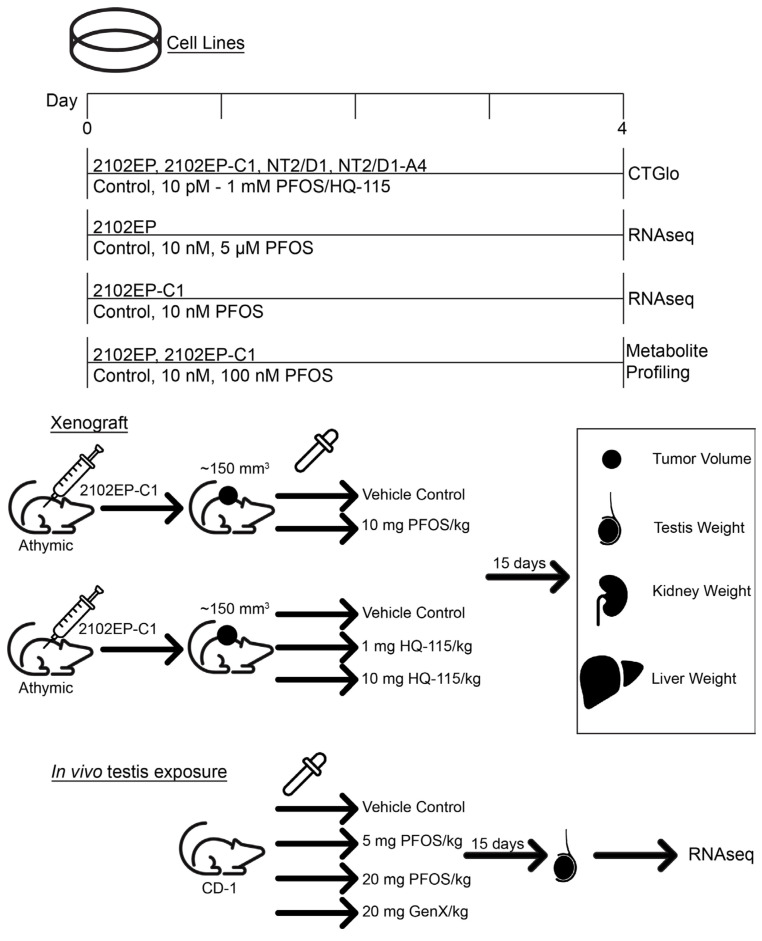
Schematic of experimental protocols.

The human embryonal carcinoma cell lines 2102EP and NT2/D1 were purchased from ATCC (Manassas, VA, USA) and authenticated by ATCC by means of karyotyping and short tandem repeat profiling. The 2102EP-C1 and NT2/D1-A4 cells are isogenic-derived cisplatin-resistant clones of 2102EP and NT2/D1 and described previously [31]. These cisplatin-resistant cells were included as we have previously shown that their cisplatin resistance is driven by epigenetic alterations which is a suspected mechanism of PFAS action [13,31]. Cisplatin is the main treatment for TGCTs and cisplatin resistance is a clinical problem [15]. Cells were maintained in Dulbecco’s Modified Eagle Medium with 10% fetal bovine serum (GeminiBio, Sacramento, CA, USA), 1% antibiotic/antimycotic (Corning, Corning, NY, USA), and 1% L-glutamine (Corning, Corning, NY, USA). For cell proliferation/survival assays, an equal number of cells were plated in 96-well plates in three biological replicates at 20,000 cells per well. On the next day, the cells were treated with PFOS (Sigma, St. Luis, MO, USA), HQ-115 (Sigma, St. Luis, MO, USA), or vehicle control (DMSO), and viable cell numbers were estimated 3 days later using the CellTiter-Glo assay (Promega, Madison, WI, USA). PFOS is a legacy PFAS and HQ-115 is a newer, “clean energy” PFAS. Both compounds were used in cell proliferation assays at doses ranging from 10 pM to 1 mM. A schematic of all experimental protocols in this proposal is provided in Figure 1.

### 2.2. Xenograft Experiments

All animal experiments were approved by the University of Illinois Urbana-Champaign IACUC under protocol 21117. For mouse studies, 5–8-week-old male athymic nude mice (Jackson Labs, Bar Harbor, ME, USA), which are immunocompromised as required for human xenograft studies [32], were injected subcutaneously in the flank with 5 × 10^6^ 2102EP-C1 cells after resuspension in a 50:50 ratio of DMEM/Matrigel (Corning, Corning, NY, USA). Once palpable tumors were detected, tumor volume was measured twice weekly with calipers using the formula V = (L × W × W)/2 [33]. At a tumor volume of approximately 150 mm^3^, mice were randomly assigned to vehicle, PFOS, or HQ-115 treatment groups. PFOS and HQ-115 were dissolved in 0.5% Tween 20 and 0.1% dimethylsulfoxide (DMSO) and orally gavaged daily for 15 consecutive days at 10 mg/kg body weight for PFOS and 1 mg/kg and 10 mg/kg body weight for HQ-115 [34]. Control mice were treated with 0.5% Tween 20 and 0.1% DMSO. Body weight was also measured twice weekly and PFAS dosing was adjusted for change in body weight. At the end of treatment, mice were sacrificed when tumors reached humane endpoints in PFAS-treated mice (approximately 20 days) with euthanasia by carbon dioxide, followed by cervical dislocation, and the testes, kidneys, and liver were harvested and weighed. Humane endpoint is defined as any tumor reaching greater that 2.0 cm in any direction [35].

### 2.3. RNA-Sequencing and Gene Set Enrichment and Gene Signature Analysis

The 2102EP cells were treated with 10 nM or 5 µM PFOS and 2102EP-C1 cells were treated with 10 nM PFOS for 4 consecutive days. RNA was extracted from cell cultures using RNeasy Mini Kit (Qiagen, Germantown, MD, USA) in biological quadruplicate. For mouse testis studies, male CD-1 mice aged 5–8 weeks old were purchased from Jackson Labs. Mice were dosed by being orally gavaged with PFOS (Sigma, St. Luis, MO, USA) and GenX (Sigma, St. Luis, MO, USA) dissolved in 0.5% Tween 20 and 0.1% DMSO daily for 15 days at 5 mg/kg and 20 mg/kg body weight for PFOS and 20 mg/kg body weight for GenX [34]. Control mice were treated with 0.5% Tween 20 and 0.1% DMSO. Whole testes were harvested in RNA-later (Invitrogen, Waltham, MA, USA), and later, RNA was isolated using the RNeasy Mini Kit in biological triplicate.

The Roy J. Carver Biotechnology Center performed RNA sequencing. RNA-seq libraries were prepared using the TruSeq Stranded mRNA Sample Prep kit. Libraries were sequenced on a HiSeq 4000 using HiSeq 4000 sequencing kit version 1. Quality controls were performed using FASTQC. Trimmomatic was used to remove low-quality bases from starts and ends with a threshold of LEADING <28 and TRAILING <28, respectively, with a minimum length of 30. The resulting clean reads were then aligned to human genome assembly NCBI GRCh38.P_12_ or mouse genome assembly NCBI GCF_000001635.27_GRCm39 using STARaligner. The resulting reads were used to count reads mapping to each gene in each sample using feature counts. All samples had comparable numbers of reads and comparable quality scores. The mean quality value across each base position was >30, representing a 99.9% base call accuracy. Differentially expressed genes were identified using the Limma Bioconductor package. The RNA-seq datasets from this study will be submitted to the NCBI Database of GEO Datasets.

Gene set enrichment analysis (GSEA) was performed using a minimum and maximum gene set size of 15 and 500, respectively. The analysis compared PFOS-treated 2102EP and 2102EP-C1 cells against their respective controls. The number of permutations was 1000, and the permutation type was gene_set. Normalized enrichment scores (NESs) indicate the distribution of Gene Ontology categories across a list of genes ranked by hypergeometrical score (HGS).

### 2.4. GC-MS Metabolite Profiling

Cells were treated with indicated doses of PFOS for 4 days. Samples were collected in 800 μL of a 3:2:3 (*v*/*v*/*v*) mixture of acetonitrile/water/isopropanol in biological triplicate. Metabolite profiling was conducted by the Carver Metabolomics Core, University of Illinois Urbana-Champaign Roy J. Carver Biotechnology Center, as previously described [36]. Each sample was analyzed using a gas chromatography–mass spectrometry (GC-MS) system consisting of an Agilent 7890 gas chromatograph (Agilent Technologies, Santa Clara, CA, USA), Agilent 5975 MSD, and HP 7683B autosampler. GC was performed on a ZB- 5MS (60 m × 0.32 mm I.D. and 0.25 μm film thickness) capillary column (Phenomenex, Torrance, CA, USA). The inlet and MS interface temperature was 250 °C, and the ion source temperature was adjusted to 230 °C. A 1 μL aliquot of sample was injected with a split ratio of 7:1. The helium carrier gas was held at a flow rate of 2 mL/min. Isothermal heating was set at 70 °C for 5 min, followed by an oven temperature increase of 5 °C/min to 310 °C, then the final 10 min at 310 °C. The MS was operated in positive electron ionization (EI) mode at 69.9 eV ionization energy with the scan range at *m*/*z* 30–800. Peaks were identified using the Automatic Mass Spectral Deconvolution and Identification System (AMDIS) v2.71 (National Institute of Standards and Technology, Gaithersburg, MD, USA) software and a custom-built MS database derived from in-house chemical standards and the National Institute of Standards and Technology (NIST) database for annotation confirmation. All data were normalized to the internal standard (hentriacontanoic acid at 10 mg/mL).

MetaboAnalyst 5.0 [37] was used for pairwise comparisons for cluster heatmap, PCA, and pathway analysis. Features with >50% missing values were removed. Variables were removed at a threshold of 50%, and missing variables were replaced with 1/5 of the minimum positive value of each variable (assumed limit of detection). Data were log transformed and auto scaled for heatmaps. For hierarchical clustering, the “hclust” function was used in the “stat” package with distance measured with “Euclidean” and clustering algorithm using “ward.D”. Quantitative-enrichment and pathway analysis was performed via metabolite set enrichment analysis (MSEA) using metabolites with KEGG-IDs using the log transformed and auto scaled data. Table S3 contains a univariate analysis and summary statistics consisting of Chemical IDs using the in-house database, metabolite classes derived from a Classy Fire, and MetaboAnalyst-generated univariate statistical results [38].

### 2.5. Statistics

Student’s *t*-tests were performed using GraphPad Prism 9 version 2. *P*-values indicative of non-significance (*p* > 0.05) and significance (*p* ≤ 0.05) were determined. Mean and standard error of the mean were used to describe sample variability.

## 3. Results

### 3.1. PFOS and HQ-115 Promote Tumor Cell Growth In Vivo

**Figure 2:**
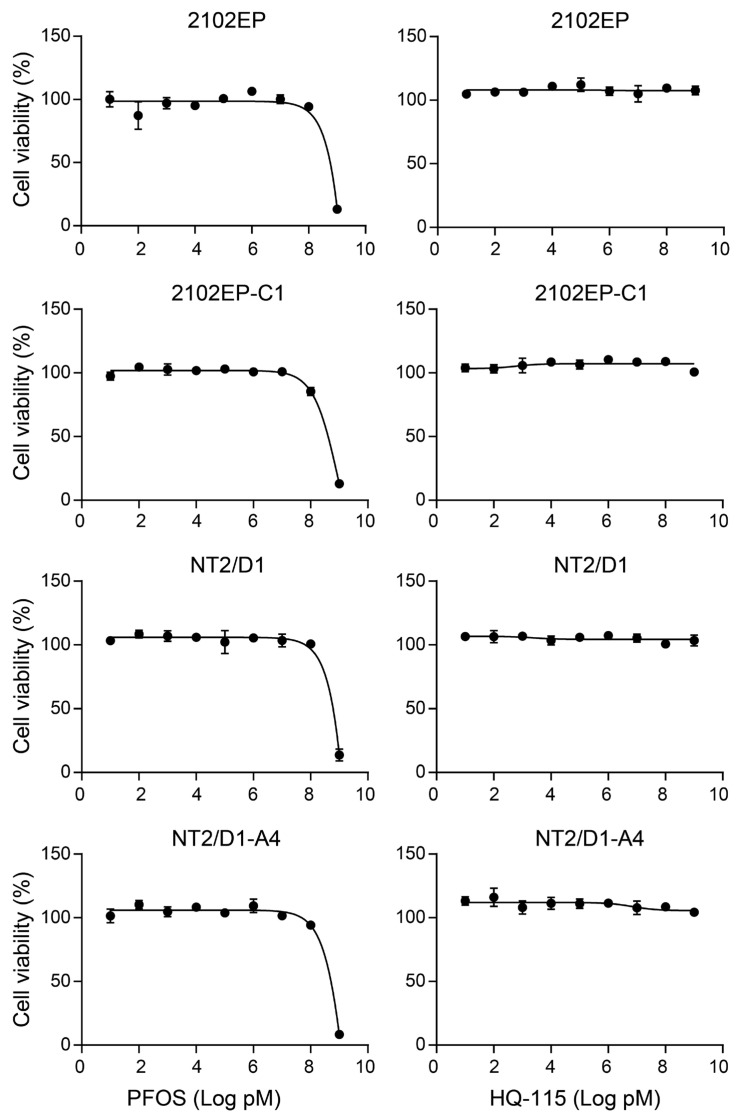
PFOS and HQ-115 are not acutely toxic and do not alter the proliferation of TGCT cells in vitro. TGCT cells lines 2102EP, 2102EP-C1, NT2/D1, and NT2/D1-A4 were treated with 10 pM to 1 mM doses of PFOS and HQ-115 for 4 days, and cell viability was assessed. Cell viability (%) is the CellTiter-Glo signal compared to untreated vehicle control. Data represent mean +/− standard error of the mean of four biological replicates, and are representative of two independent experiments.

To begin to investigate the effects of PFAS chemicals on TGCT tumorigenic properties, we conducted proliferation and survival assays on parental TGCT cells 2012EP and NT2/D1 and derived cisplatin-resistant cells 2102EP-C1 and NT2/D1-A4 treated with PFOS and HQ-115 (Figure 2). A wide range of doses from 10 pM to 1 mM was chosen to cover the full range of human PFOS exposures. According to the Agency of Toxic Substances and Disease Registry, PFOS mean human serum levels for the general population, contaminated communities, and the occupationally exposed are 8 nM, 20 nM, and 1.9 µM, respectively [39]. Consensus-relevant human doses for HQ-115 are still to be defined. Over this large dose range, both PFOS and HQ-115 had little effect on cell proliferation and the survival of TGCT cells over a 4-day exposure window, suggesting that PFOS and HQ-115 are not acutely toxic to TGCT cells (Figure 2). It should be noted that the highest, 1 mM dose of PFOS, but not the 1 mM dose of HQ-115, was acutely toxic to all four cell lines; however, this dose of PFOS is not realistically achievable in humans [39].

**Figure 3:**
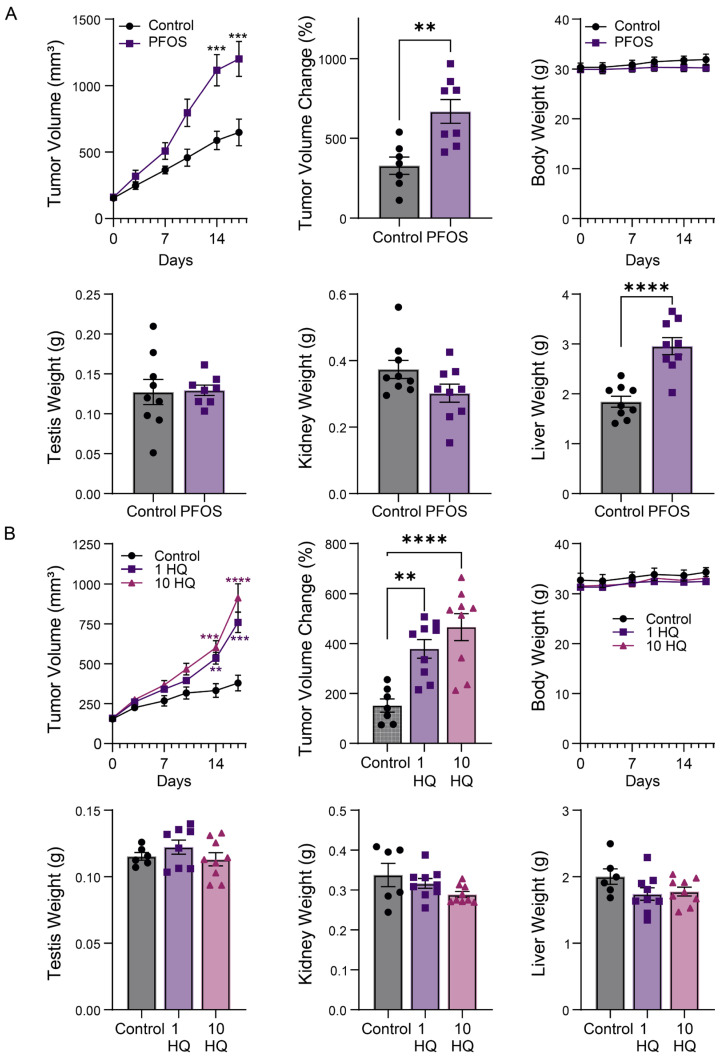
PFOS and HQ-115 promote TGCT growth in vivo. (**A**) TGCT xenograft (as described in Materials and Methods) of 2102EP-C1 cells treated for 15 consecutive days with 10 mg/kg PFOS or vehicle control. Tumor volume over time, percent tumor volume change (from day 1 of treatment to day 15 of treatment), and testis, kidney, and liver weight are presented. Data represent mean +/− standard error of the mean, and are representative of two independent experiments. ** *p* ≤ 0.01, *** *p* ≤0.005, **** *p* ≤ 0.001. (**B**) TGCT xenograft of 2102EP-C1 cells treated for 15 days with 1 mg/kg (1 HQ in figure) and 10 mg/kg HQ-115 (10 HQ in figure) or vehicle control. Tumor volume over time, percent tumor volume change (from day 1 of treatment to day 15 of treatment), and testis, kidney and liver weight are presented. Data represent mean +/− standard error of the mean. ** *p* ≤ 0.01, *** *p* ≤0.005, **** *p* ≤ 0.001.

To better define the effects of PFAS on TGCTs tumorigenicity, the effects of PFOS and HQ-115 on TGCT progression in vivo were assessed. Immunocompromised mice were engrafted with 2101EP-C1 cells and treated orally with the vehicle control, PFOS, or HQ-115 for 15 days. PFOS at 10 mg/kg significantly increased the progression of testicular tumors over control-treated mice (Figure 3). This dose of PFOS did not affect overall body, kidney, or testis weight, but did significantly increase liver weight, suggesting that PFOS may have toxic effects on the liver under these conditions. The effects of HQ-115 on TGCT progression were tested at two doses, 1 mg/kg and 10 mg/kg. Both doses of HQ-115 also promoted the growth of testicular tumors over control-treated mice (Figure 3). HQ-115 did not affect body, testis, and kidney weight and, in contrast to PFOS, did not affect liver weight either. This result is in line with the above in vitro experiments, suggesting that HQ-115 may be less toxic than PFOS and that liver toxicity is not associated with the growth effects of HQ-115 on testicular tumors.

### 3.2. PFOS and GenX Alter Gene Expression Associated with Fatty Acid and Steroid Synthesis and Metabolism, Epigenetics, and Other Pathways Associated with Pro-Cancer Phenotypes in Mouse Testes and Human TGCT Cells

**Figure 4:**
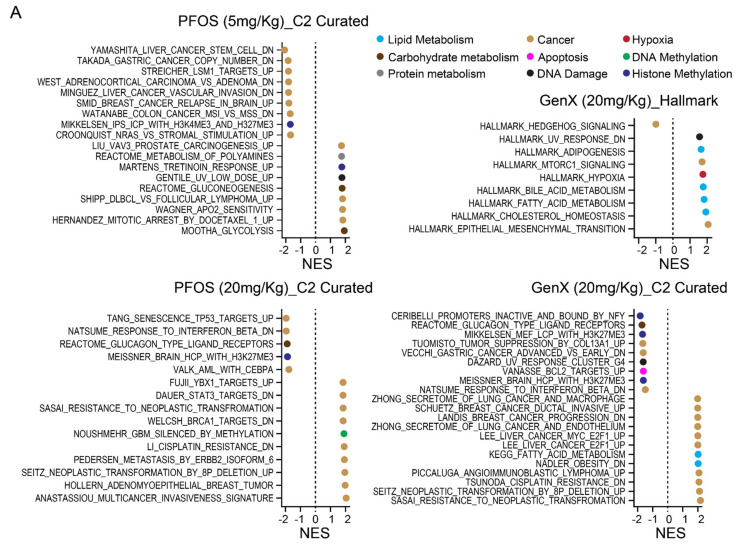

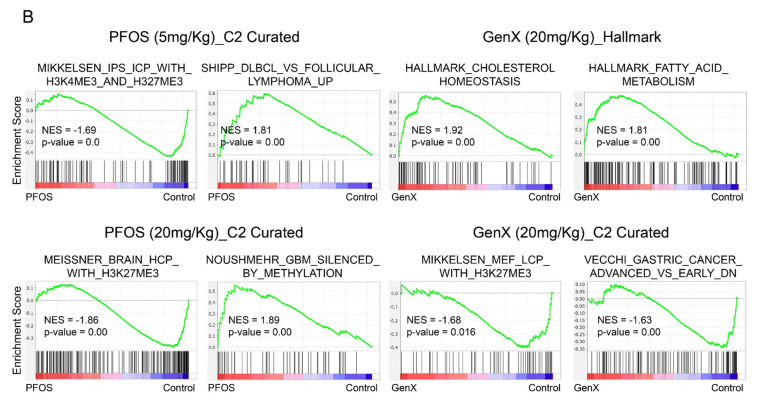
PFOS and GenX alter gene expression associated with fatty acid and steroid synthesis and metabolism, H3K27me3, DNA methylation, and other pathways associated with pro-cancer phenotypes in mouse testes. Results of the RNA-seq analysis of mouse testes after oral treatment with 5 mg/kg and 20 mg/kg per body weight of PFOS or 20 mg/kg per body weight of GenX for 15 consecutive days compared to vehicle control. (**A**) Gene set enrichment analysis (GSEA) of gene sets among the top 15 ranked by normalized enrichment score (NES) altered by PFOS or GenX treatments. Complete lists are in Supplemental Table S1. (**B**) Representative gene set enrichment plots.

We performed an RNA-seq expression analysis of mouse testes after 15-day daily oral dosing of PFOS at 5 mg/kg and 20 mg/kg body weight. We also performed RNA-seq on mouse testes after 15-day daily oral dosing with the second-generation PFAS, GenX. Robust and consistent transcriptional changes were evident in PFAS-treated testes compared to control testes. We then performed gene set enrichment analysis (GSEA) against the C2 gene sets from the MSigDB database. Within the top 15 PFOS and GenX up- and downregulated gene sets, as determined by normalized enrichment score (NES), PFOS and GenX treatments consistently downregulated gene sets associated with the H3K27me3 mediated polycomb pathway and altered gene sets associated with DNA methylation (Figure 4 and Supplemental Table S1). We have previously shown that the polycomb pathway and DNA methylation are interconnected and reciprocally regulated epigenetic pathways associated with the survival of TGCT cells under stress conditions [31,40,41]. Several other cancer-related pathways were altered by PFOS and GenX within the top 15 regulated gene sets including those involved in hypoxia, apoptosis, DNA damage, and cancer tumorigenesis and metastasis (Figure 4 and Supplemental Table S1). Interestingly, alterations in gene sets associated with fatty acid and cholesterol metabolism were seen especially with GenX when assessing the MSigDB Hallmark database (Figure 4 and Supplemental Table S1). Previously, PFAS have been suggested to function as fatty acid mimics based on their similarity of structure, and regulate fatty acid steroid metabolism including acting as ligands for peroxisome proliferator-activated receptors (PPARs) [7,42,43].

**Figure 5:**
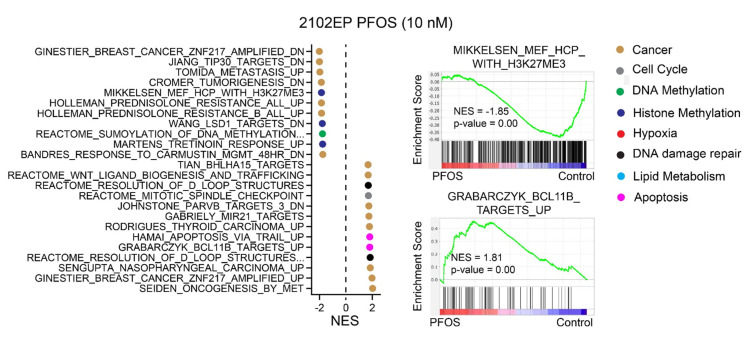

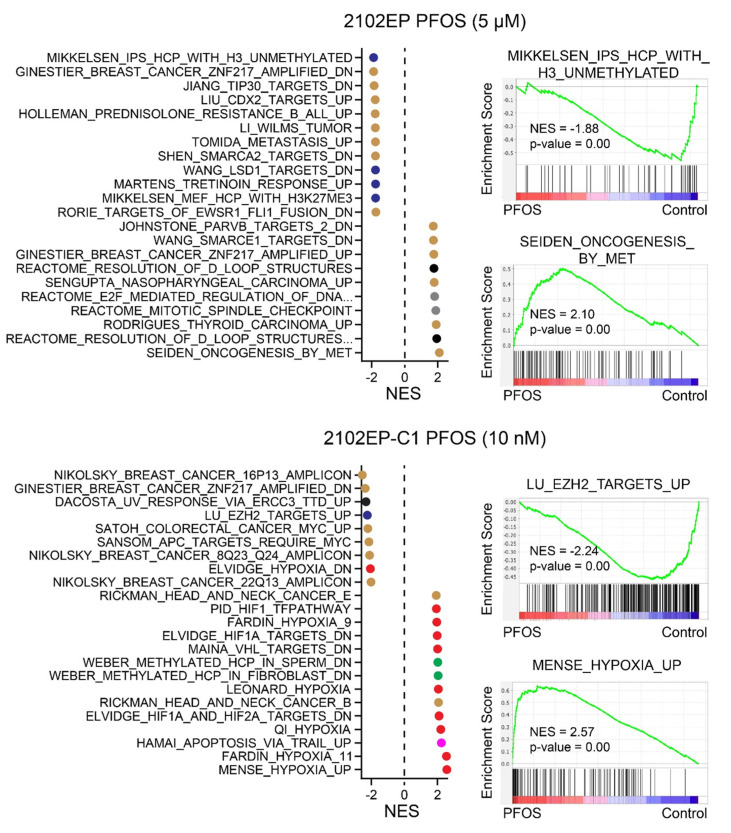
PFOS alters gene expression associated with H3K27me3, DNA methylation, and other pathways associated with pro-cancer phenotypes in human TGCT cells. Results of the RNA-seq analysis of 2102EP TGCT cells treated with 10 nM or 5 µM PFOS and 2012EP-C1 TGCT cells treated with 10 nM PFOS for 4 days compared to vehicle control. Gene set enrichment analysis (GSEA) showing gene sets and representative gene set enrichment plots among the top 15 ranked by normalized enrichment score (NES) altered by PFOS. Complete lists are in Supplemental Table S2.

A transcriptomic analysis was also performed with 2102EP cells treated acutely with 10 nM and 5 µM PFOS and 2102EP-C1 cells treated with 10 nM PFOS for 4 days in vitro. Similar to the PFAS-mediated changes in mouse testes, there were a consistent downregulation of gene sets associated with the H3K27me3 mediated polycomb pathway and an alteration in gene sets associated with DNA methylation, as well as alterations in cancer pathways associated with tumorigenesis, apoptosis, hypoxia, and DNA repair (Figure 5 and Supplemental Table S2).

### 3.3. PFOS Alters the Metabolomic Profile of TGCT Cells Including Prominent Regulation of Steroid Biosynthetic and Fatty Acid Homeostasis-Associated Metabolites

**Figure 6:**
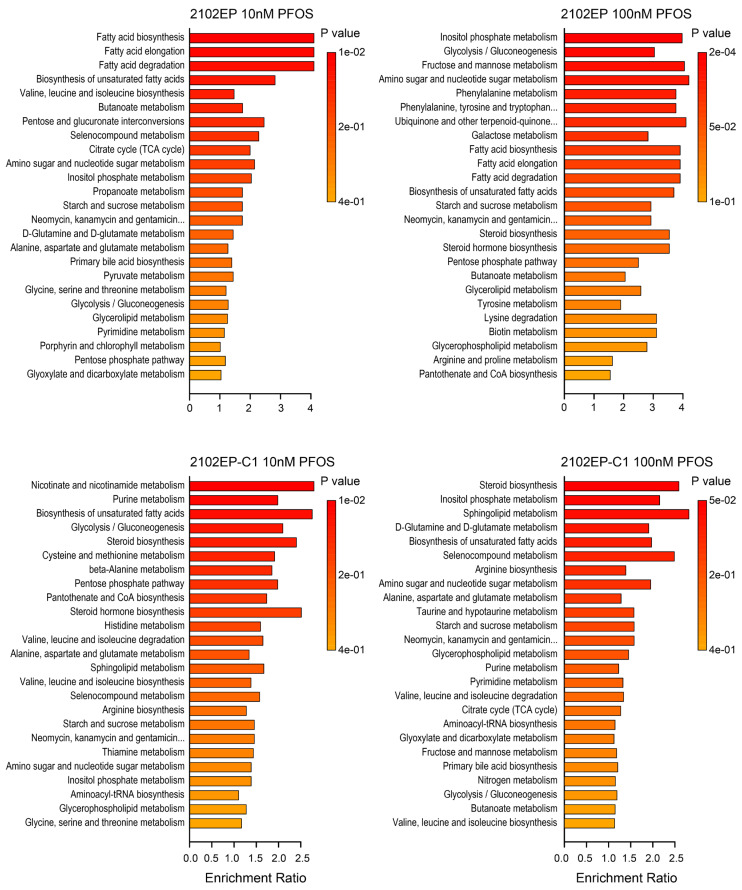
PFOS alters metabolite profiles of TGCT cells. GC-MS metabolite profiling of 2102EP and 2102EP-C1 cells treated for 4 days with 10 nM and 100 nM PFOS. Enrichment pathway analysis was performed with MetaboAnalyst 5.0.

**Figure 7:**
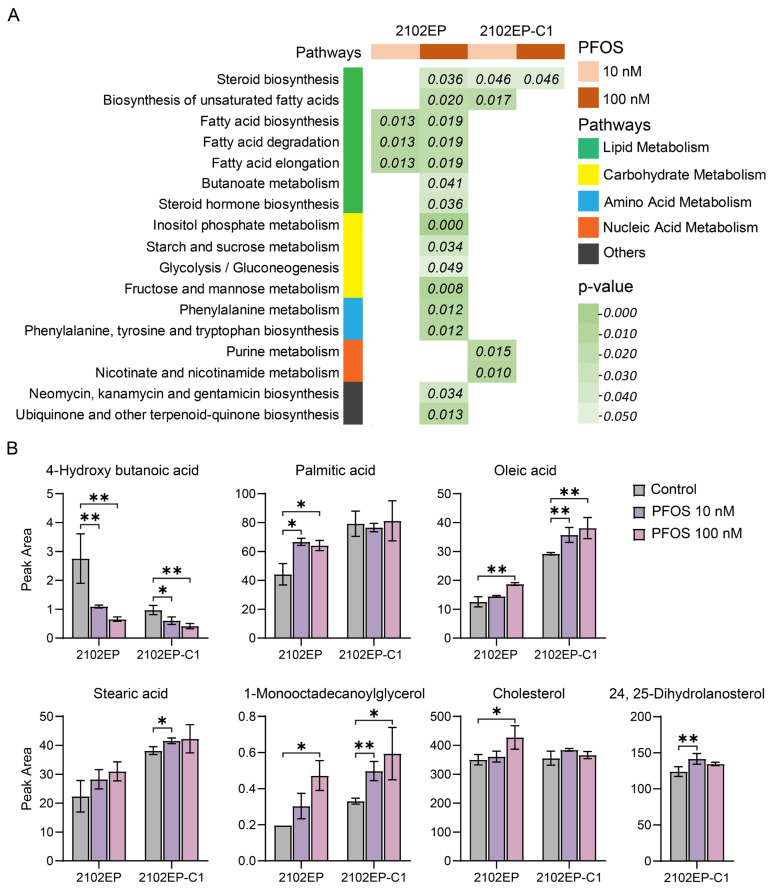
PFOS alters metabolite products associated with fatty acid and steroid biosynthesis in TGCT cells. 2102EP and 2102EP-C1 cells were treated for 4 days with 10 nM and 100 nM PFOS. (**A**) Compilation of most commonly significantly enriched metabolic pathways as performed with MetaboAnalyst 5.0 across cell lines and treatments. (**B**) Univariate analysis demonstrating significantly altered fatty acid-related metabolites upon PFOS treatments. Metabolite units are in peak area adjusted for internal standard. Data represent mean +/− standard error of the mean. * *p* ≤ 0.05, ** *p* ≤ 0.01.

A GC-MS metabolite profiling analysis was then conducted on 2102EP and 2102EP-C1 cells treated with 10 nm and 100 nm of PFOS for 4 days. The principal component analysis (PCA) clearly separated PFOS-treated biological replicates from controls (Supplemental Figure S1). Several metabolites were substantially and consistently altered in PFOS-treated cells compared to controls, many of which were related to fatty acid metabolism, including 4-hydoxybutanoic acid, palmitic acid, oleic acid, 1-monooctadecanoylglycerol, and stearic acid (Supplemental Figure S2 and Table S3). Since PFOS and other PFAS are suspected fatty acid mimics and proposed ligands for nuclear receptors that regulate fatty acid metabolism including PPAR-α, we next performed a pathway enrichment analysis [7,42,43]. In agreement with our transcriptomic results, the most significant and consistent pathways regulated by PFOS in TGCT cells were related to fatty acid homeostasis and steroid biosynthesis (Figure 6 and Figure 7, Supplemental Table S4). Together, the data suggest that the pro-cancer effects of PFOS on TGCTs may in part be mediated by the cell-intrinsic regulation of fatty acid metabolism.

## 4. Discussion

Although there is a consistent link between PFAS exposure and cancer, there are limited studies on its mechanism. PFAS are non-mutagenic [13]. Hence, it is logical that epigenetic mechanisms may be key drivers of increased cancer risk with PFAS exposure. Epidemiologic evidence has linked PFAS to TGCTs [7]. The known etiology of TGCTs strongly suggests that compared to most solid tumors, epigenetics play an especially prominent role in TGCT biology [15,16,17,18,19,20,21]. Hence, TGCTs may be an especially sensitive tumor type to uncover mechanisms of PFAS and cancer. Ours is the first investigation to directly address the biological effects of PFAS on TGCTs. In this report, the effects of the legacy PFAS PFOS and clean energy PFAS HQ-115 on the tumorigenicity of TGCTs in mice, TGCT cell survival and metabolite production, as well as gene regulation were investigated. PFOS and HQ-115 promoted TGCT growth in mice, but not in the cell culture, suggesting that PFAS does not directly affect TGCT proliferation but rather alters the in vivo tumorigenicity of TGCT cells. This pro-cancer phenotype was linked to alterations in several cancer-related pathways. Metabolomic studies revealed that PFOS also altered several metabolite products including those associated with steroid biosynthesis and fatty acid metabolism in TGCT cells, consistent with the proposed ability of PFAS to mimic fatty acid and the proposed role of PFAS as endocrine disrupters.

Testicular cancer has one of the strongest epidemiological links to PFAS exposure, including cohort and ecological/case–control studies [24,25,26,27,28,29,30]. In the C8 Health Project Dupont plant study of individuals in a community exposed from 1950 to 2004, the incidence of testicular cancer increased with increasing PFOA serum levels, with a 3-fold higher risk in the most exposed group [28]. A scoping review of 16 cohort studies, 10 case–control studies, 1 cross-sectional study, and 1 ecological study concluded that the cancer sites with the most compelling evidence for an association with PFAS exposure across studies were kidney and testicular cancers [27]. A separate meta-analysis, focused on kidney and testicular cancer, indicated a significant increase in TGCT risk per 10 ng/mL increase in serum PFOA, and that this association was most likely causal [30]. Finally, a recent nested case–control study found increased TGCT relative risks with elevated PFOS concentrations in firefighting Air Force servicemen [25].

An interesting finding of our work was the link between PFOS exposure in mouse testes and human TGCT cells and metabolite products associated with fatty acid metabolism and steroid biosynthesis, which is consistent with the known fatty acid mimicry properties of PFAS [7,42,43]. Testicular cancer is a disease of developmental origin, arising from aberrant primordial germ cells/gonocytes in utero [15,17]. Epidemiologic studies have indicated that the fetal gonads may be especially sensitive to pro-estrogenic and anti-androgenic insults [18,19,20,21]. For example, a meta-analysis of 10 studies on endocrine disrupting chemicals (EDCs) and testicular cancer risk concluded that maternal exposure to EDCs was associated with a >2-fold higher risk of testicular cancer in offspring [20]. This has led to the theory that TGCTs can be viewed as part of a continuum on a “testicular dysgenesis syndrome” spectrum that includes cryptorchidism, hypospadias, poor semen quality, and male subfertility due to environmental perturbations, especially those associated with low androgen levels during gonadal development [18,19,44,45].

In turn, several studies in mice and humans suggest an increase in male reproductive toxicities after prenatal PFAS exposure [46,47,48]. These include adverse effects on semen quality and quantity, and high estrogen/low testosterone levels, which are known to be risk factors for human TGCTs [49,50,51]. Hence, it is tempting to speculate that alterations in steroidogenesis by in utero PFAS exposure may lead to an increased TGCT risk in male offspring. This fatty acid mimicry effect of PFAS may also be related to the liver toxicity that was noted with PFOS treatments in our studies as the liver is a major organ of fatty acid metabolism. It would be of interest to directly dissect the precise role, if any, of PPARs in mediating PFOS and HQ-115 effects on TGCTs. 

Of particular interest to us was the PFOS regulation of the epigenetic pathways of H3K27me3/polycomb and DNA methylation in mouse testes and human TGCT cells. TGCTs are highly histologically heterogeneous despite having a uniform cytogenic and genomic profile [15,52]. Further, TGCTs have few mutations [16]. This suggests that epigenetic mechanisms play an important role in the etiology and biology of TGCTs [15,17]. Notably, we previously established epigenetic remodeling via the polycomb pathway and DNA methylation as important regulators of TGCT biology [31,40,41]. Hence, the PFAS-mediated epigenetic remodeling of TGCT cells may be a second mechanism to account for our observed increase in tumor growth with PFOS. Further, because TGCTs appear highly sensitive to environmental exposures during development, they may be a particularly relevant model to study the impact of PFAS on health outcomes.

## 5. Conclusions

Testicular germ cell cancer is perhaps the strongest epidemiological link to PFAS exposure, yet there have been no biological studies. The current report is the first study to assess biological effects of PFAS chemicals on TGCTs. We found that PFOS and HQ-115 promoted the in vivo growth of TGCTs and the pro-tumor effect of PFOS was associated with consistent transcriptional changes in several pro-cancer pathways, especially polycomb, DNA methylation, and fatty acid and steroid metabolism pathways. In addition, PFOS altered metabolite products associated with fatty acid and steroid biosynthesis. We speculate that TGCTs may be a sentinel cancer type for PFAS exposure. 

## Data Availability

The RNA-seq datasets for the current study have been submitted to the NCBI Database of GEO Datasets under the accession number GSE262137.

## Supplementary Materials

The following supporting information can be downloaded at: https://www.mdpi.com/article/10.3390/toxics12040232/s1, Figure S1. Principal component analysis of the metabolic profile of TGCT cells treated with PFOS. EP, 2102EP; C, 2102EP-C1; 10, 10 nM PFOS; 100, 100 nM PFOS; control, vehicle control. 2102EP and 2102EP-C1 cells treated for 4 days with 10 nM and 100 nM PFOS. Figure S2. Hierarchical clustering of metabolites altered in TGCT cells treated with PFOS. EP, 2102EP; C, 2102EP-C1; 10, 10 nM PFOS; 100, 100 nM PFOS; control, vehicle control. 2102EP and 2102EP-C1 cells treated for 4 days with 10 nM and 100 nM PFOS. Table S1. GSEA_Mouse Testis. Top 15 up- and downregulated gene expression pathways after PFOS and GenX treatments of mice. Mice were orally treated with 5 mg/kg and 20 mg/kg per body weight of PFOS or 20 mg/kg per body weight of GenX for 15 consecutive days. Table S2. GSEA_TGCT cells. Top 15 up- and downregulated gene expression pathways after PFOS treatment of TGCT cells. 2102EP TGCT cells were treated with 10 nM or 5 µM PFOS and 2012EP-C1 TGCT cells were treated with 10 nM PFOS for 4 days. Table S3. Metabolomic univariate analysis. Univariate analysis and summary statistics of GS-MS metabolomic analysis of PFOS-treated TGCT cells. 2102EP and 2102EP-C1 cells were treated for 4 days with 10 nM and 100 nM PFOS. Table S4. Metabolomic pathway enrichment. Compilation of top enriched pathways of the GS-MS metabolomic analysis of PFOS-treated TGCT cells performed with MetaboAnalyst. 2102EP and 2102EP-C1 cells were treated for 4 days with 10 nM and 100 nM PFOS.
