# Supplementary figures and images for: Perfluorooctanesulfonic Acid Alters Pro-Cancer Phenotypes and Metabolic and Transcriptional Signatures in Testicular Germ Cell Tumors
Source: Toxics. 2024 Mar 22;12(4):232. doi: 10.3390/toxics12040232 (PMC11054796; doi:10.3390/toxics12040232)

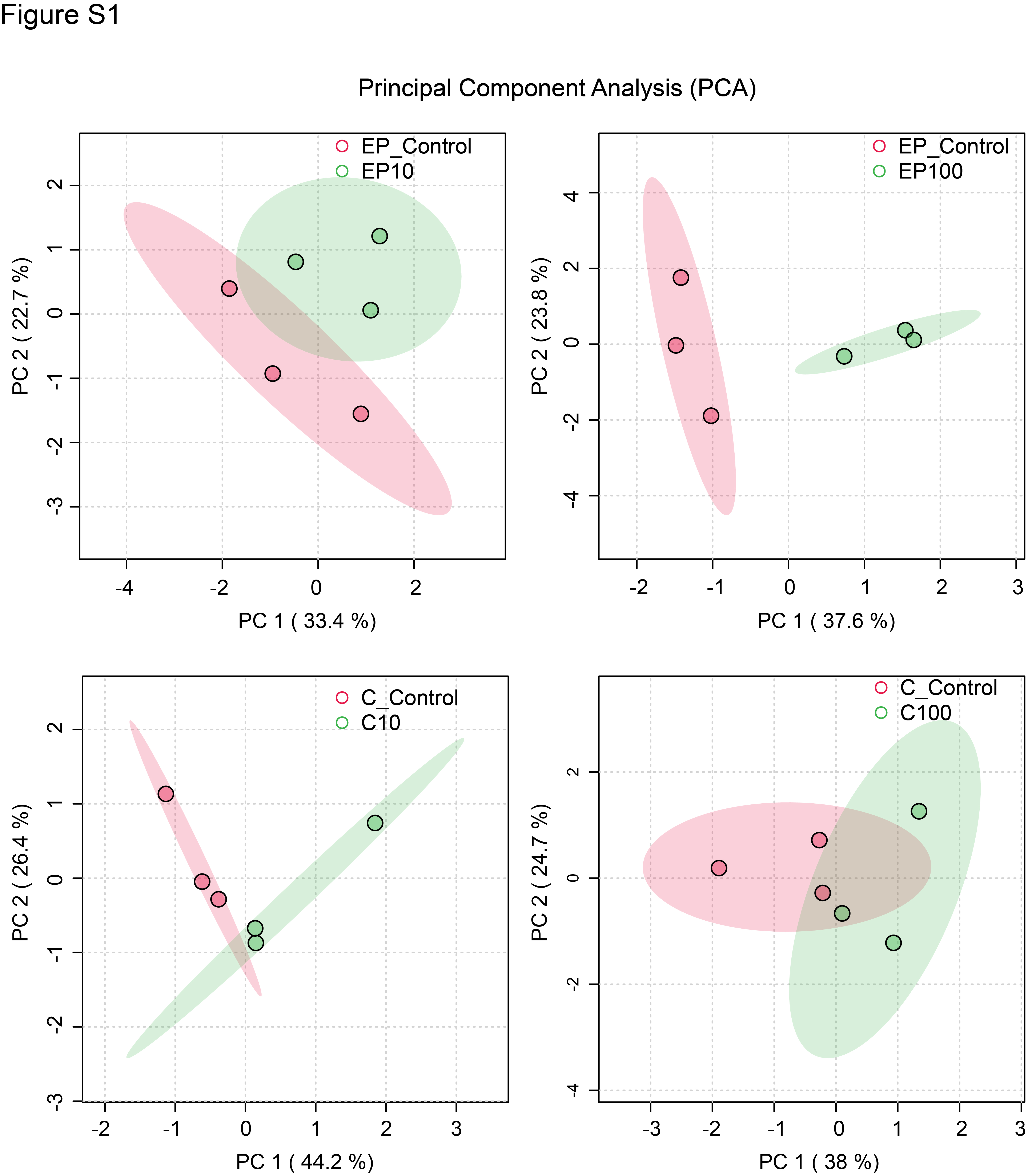

Supplement: Supplementary file 1 [file toxics-12-00232-s001.zip › Manuscript supplemental/Figure S1.jpg]

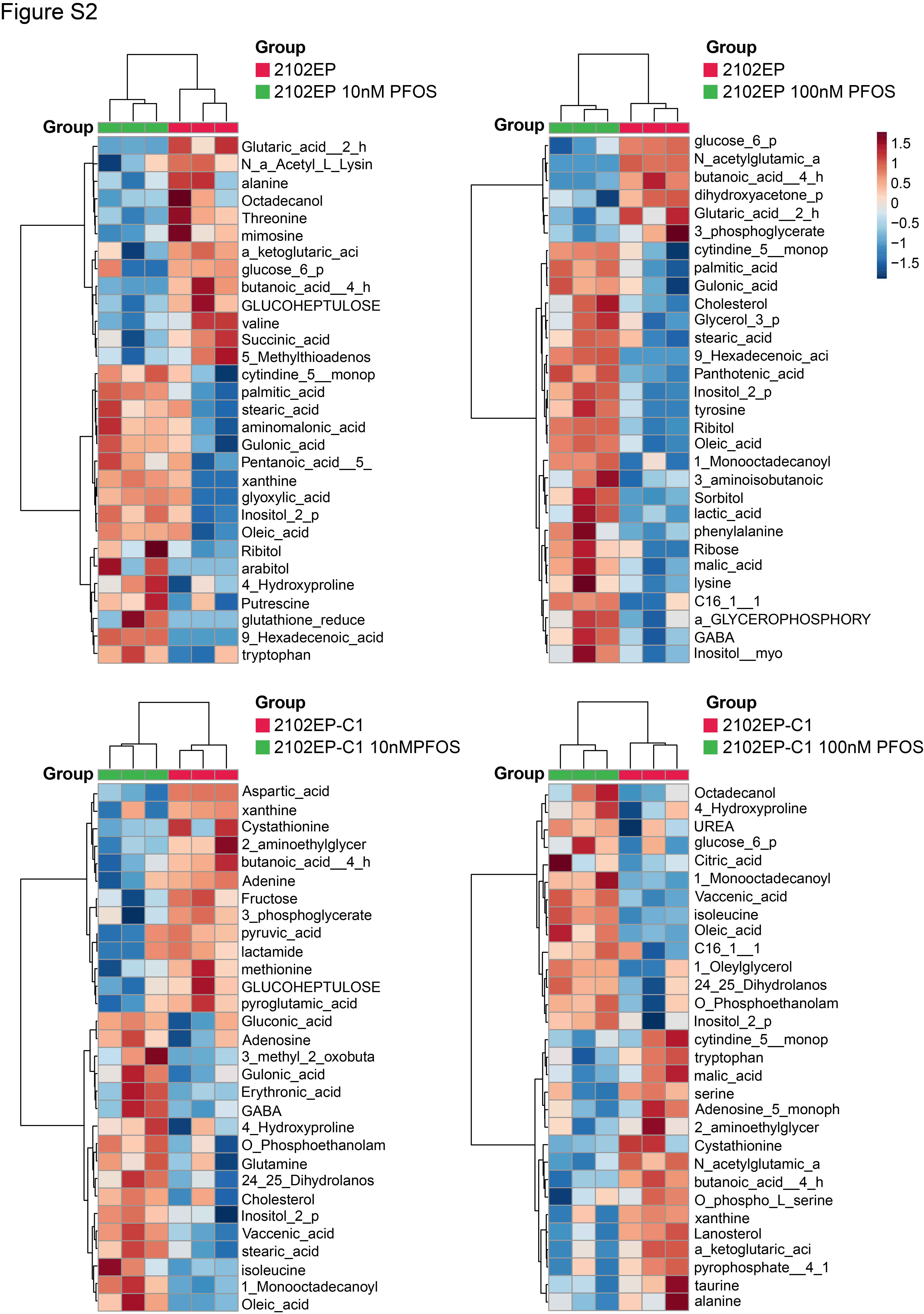

Supplement: Supplementary file 1 [file toxics-12-00232-s001.zip › Manuscript supplemental/Figure_S2.jpg]
